# Omentin level in serum and gingival crevicular fluid following non-surgical periodontal therapy among Rheumatoid Arthritis patients

**DOI:** 10.6026/973206300210623

**Published:** 2025-04-30

**Authors:** Geetha Ari, Jyothi Mohit, Komal Vilas, Thirumagal K., Vaishali Kamak R., Anitha Logaranjani, Jaideep Mahendra

**Affiliations:** 1 Department of Periodontics, Meenakshi Ammal Dental College and Hospital, Meenakshi Academy of Higher Education & Research (MAHER), Chennai, Tamil Nadu, India; 2 Private practitioner, Smile Craft Dental Care, Chennai, Tamil Nadu, India

**Keywords:** Omentin, rheumatoid arthritis, non-surgical periodontal therapy

## Abstract

Non-surgical periodontal therapy on serum and gingival crevicular fluid levels of omentin among periodontitis subjects with and without Rheumatoid Arthritis is of interest. Hence, 33 patients were selected and divided into three groups: P group, RA group and RA+P group. Periodontal parameters were recorded; samples of serum and gingival crevicular fluid were obtained to evaluate Omentin levels. All of them showed non-surgical periodontal therapy and were recalled after 6 weeks. The mean change in periodontal parameters from T0 to T1 was statistically significant but serum and gingival crevicular fluid levels of omentin were not statistically significant for considering it as an anti-inflammatory marker.

## Background:

One of the most prevalent inflammatory conditions is periodontitis, which causes significant harm to the tissues that support teeth and eventually leads in tooth loss. Like many other systemic inflammatory illnesses, periodontitis also leads to complicated interaction between the periodontal pathogens and the immune system. This leads to the release of many inflammatory cytokines like interleukin -1b, TNF-a and many others [1]. Rheumatoid arthritis (RA) is also an inflammatory disease of the joints caused due to autoimmune attack of periarticular tissues. The etiology of RA has been clarified and the role of peptide citrullination as an etiopathologic event has been well elucidated. Peptidylarginine deaminase (PAD) is an enzyme which can cause posttranslational modification and neoepitope generation of proteins with subsequent development of anticitrullinated peptide antibodies (ACPA) [2]. A number of hypothesized interactions between periodontal disease and rheumatoid arthritis were proposed. Some of them include inflammatory disease pathways shared risk factors such as smoking and difficulty in maintaining oral hygiene due to temporomandibular joint and/or peripheral joint dysfunction. Later, it was discovered that the major bacterial species, Porphyromonas gingivalis, involved in the pathogenesis of periodontal diseases, has a peptidylarginine deiminase (PAD) which is capable of citrullination. Additionally, it is widely known that inflammatory mediators play a part in the pathophysiology of both disorders [3]. Visceral adipose tissue cells release omentin, an adipokine that weighs 40 kDa and is composed of 313 amino acids. It has been described as an anti-inflammatory adipokine because of its many biological characteristics, including anti-inflammatory, antidiabetic and antiatherogenic effects [1]. Diabetes is linked to elevated omentin levels, indicating its wider relevance in inflammatory illnesses such as chronic periodontitis (CP) and others. This connection points to omentin's potential as a biomarker for monitoring inflammation in various medical conditions [2]. In our previous study, we evaluated the levels of omentin in the blood and gingival crevicular fluid (GCF) of healthy individuals, periodontitis patients and rheumatoid arthritis patients with periodontitis and without periodontitis [3]. The mean values of gingival crevicular fluid omentin and serum were statistically lower in rheumatoid arthritis with periodontitis group compared to the other three groups.

The "gold standard" for treating periodontal disease is still non-surgical periodontal therapy (NSPT), which significantly improves clinical and biochemical parameters [4]. Ding et al evaluated the effect of non-surgical periodontal therapy on the clinical features and serological parameters of patients suffering from rheumatoid arthritis as well as chronic periodontitis. He concluded that non-surgical periodontal therapy improved the oral and inflammatory condition of periodontitis patients with Rheumatoid arthritis [5]. However, this study was limited to the identification of omentin in serum samples. The identification of omentin from the oral fluid and comparing it with the serum levels following non-surgical periodontal therapy still remain unexplored as this would further help in analysing and comparing the anti-inflammatory effect of the biomarker in both the biological fluids. Therefore, it is of interest to evaluate the effect of non-surgical periodontal therapy on serum and gingival crevicular fluid levels of omentin in rheumatoid arthritis patients with and without periodontal disease.

## Materials and Methods:

## Research design:

The current cross-sectional study was conducted in Chennai, Tamil Nadu, India, between December 2022 to April 2023. Out of the fifty-five individuals that were chosen for the study, 10 individuals were removed because they had not consented to participate and 12 had associated systemic disorders that prevented them from being included in the study. Ultimately, 33 participants were chosen from the Tertiary Medical Center in Ambattur, Chennai and the Department of Periodontology at Meenakshi Ammal Dental College and Hospital in Chennai. The healthy group was left out of the study since they don't need non-surgical periodontal care, but the same patients from our earlier research were included. The Meenakshi Academy of Higher Education and Research's institutional ethical committee in Chennai gave its approval to the study (MADC/IEC-I/024-A/2021). All study participants gave their written informed permission after being fully briefed about the study's methodology.

The study was conducted in compliance with the 2013 revision of the 1975 Helsinki Declaration. Three groups were created from the study population: According to the American College of Rheumatology/European League against Rheumatism (ACR/EULAR) 2010 classification, the P group comprised 11 systemically healthy subjects with periodontitis and the RA group comprised 11 periodontally healthy subjects with rheumatoid arthritis, as determined by a rheumatologist. 11 individuals with rheumatoid arthritis and periodontitis made up the RA+P group.

The study population had to meet three requirements in order to be included: (1) be between the ages of 35-65 years; (2) individuals who are ready to participate in the study; and (3) have at least 20 natural teeth left. Furthermore, the 2017 classification of periodontal and peri-implant disease served as the basis for the identification of periodontitis groups. Both the P and RA+P groups had moderate ridge defect, class II or III furcation, vertical bone loss > 3 mm, interdental CAL greater than or equal to 5 mm, tooth loss less than 4 teeth, probing pocket greater than or equal to 6 mm and stage III grade B periodontitis.

Individuals with a history of antibiotics, steroids, immunosuppressive drugs, aspirin, or anticoagulants within the previous three months, on-going infectious diseases, pregnant or lactating women, patients with diabetes mellitus, other autoimmune diseases for RA patients, obese people, neoplasia, radiation therapy, osteoporosis, any systemic disorders that can affect adipokine levels, periodontal therapy within the previous six months, orthodontic therapy and current or past smokers were excluded from all three groups in the study.

## Parameters assessed:

Two calibrated examiners (G.A, K.V) used the University of North Carolina-15 (UNC-15) probe to record periodontal data. The following periodontal parameters were evaluated at baseline (T0): (1) Plaque Index [6] (2) Modified Gingival Index [7] (3) Probing Pocket Depth (PPD); (4) Clinical Attachment Level (CAL). Six distinct locations for each tooth - mesiofacial, facial, distofacial, mesiolingual, lingual and distolingual were used to record PPD and CAL.

## Collection of serum sample:

A 20-gauge needle and a 5-milliliter syringe were used to venipuncture each participant in each group to obtain two milliliters of venous blood from the antecubital fossa. The drawn blood sample was left at room temperature for an hour. The serum was then promptly transferred to an Eppendorf tube after being separated from the blood by centrifugation at 3000 rpm for 15 minutes at 40°C. Prior to the assay, it was kept at -80°C.

## Collection of gingival crevicular fluid sample:

To prevent blood contamination of the samples, gingival crevicular fluid (GCF) was obtained on a day other than the day of the clinical evaluation. The site with the highest score-that is, the site with the greatest amount of attachment loss was chosen to collect the gingival crevicular fluid sample. Sampling from the mesio-buccal region of the maxillary right first molar was predetermined in the healthy group (H group) in order to standardize site selection. Before the fluid was collected using a microcapillary pipette, the region was properly dried and isolated using cotton rolls. The pipette was gently positioned at the gingival sulcus entrance. For little more than fifteen to twenty minutes, each sample was taken. Samples tainted with blood or saliva was thrown away and new samples were taken ten to fifteen minutes later. Following collection, were then transferred to the sterile vials and kept at-80°C until analysis.

## Non-surgical periodontal therapy:

Using ultrasonic scalers (WoodpeckerTM, Guilin, China), P and RA+P patients received extensive subgingival scaling and root planing. Using 4R-4L or 2R-2L Columbia universal curettes (Hu-Friedy, Chicago, USA), root planing was done according to standard procedures. Everyone received oral hygiene instruction, with special attention to the use of the modified bass method, tooth brushing regularly and appropriate devices for interdental cleaning, including dental floss and interdental brushes. After six weeks (T1), they were recalled to re-evaluate the periodontal parameters and collect blood and gingival crevicular fluid.

## Omentin estimation using ELISA:

In accordance with the manufacturer's instructions, serum and gingival crevicular fluid samples were examined for omentin using the human omentin enzyme-linked immunosorbent assay (ELISA) kit (Abbkine). An ELISA plate reader (LABSERV) at 450 nm was used to measure the optical density (OD value) of each well simultaneously. Omentin has a detection range of 0.63-40 ng/mL (Figure 1 - see PDF).

## Statistical analysis:

SPSS software (IBM SPSS Statistics for Windows, Version 26.0, Armonk, NY: IBM Corp. Released 2019) was used to do statistical analysis. The results of the Shapiro-Wilks and Kolmogorov-Smirnov tests of normality showed that every variable had a normal distribution. Consequently, parametric approaches were used to analyze the data. One-way ANOVA was used to compare the mean and standard deviation for each of the three groups. Multiple pairwise comparisons between the groups were performed using Tukey's HSD post hoc test. To evaluate the linear association between omentin and periodontal parameters, the Karl Pearson correlation was used. The threshold for significance was set at 5%. (Statistically significant if the P-Value is less than 0.05).

## Results:

At baseline (T0), Plaque index, modified gingival index, probing pocket depth and clinical attachment level mean ± SD were all higher in the P & RA+P group than in the RA group (p = 0.001). However, at baseline (T0), the RA+P group had lower mean ± SD of serum and gingival crevicular fluid levels of omentin than the P and RA group (p = 0.001) (Table 1). Patients in Group P and RA+P, underwent NSPT and were reevaluated after 6 weeks (T1). At T1, the mean ± SD of the clinical attachment level, probing pocket depth, modified gingival index and plaque index decreased and plaque index and probing pocket depth were statistically significant in P and RA+P group. At T1, the mean ± SD of Serum and gingival crevicular fluid Omentin increased in both the groups and the increase was statistically significant in relation to gingival crevicular fluid Omentin (Table 2). The mean change in periodontal parameters from T0 to T1 was statistically significant but biochemical parameters like gingival crevicular fluid levels and serum of omentin was not statistically significant (Table 3). There was a substantial positive link between lower levels of omentin and more periodontal deterioration when serum and gingival crevicular fluid levels of omentin were compared with plaque index, modified gingival index, probing pocket depth and clinical attachment level in both the P and RAP+P groups (Table 4 and Table 5).

## Discussion:

The dental health of rheumatoid arthritis patients has drawn more attention in recent years, especially in light of periodontal disease. Several large epidemiological studies, as well as smaller case-control and cohort studies, have demonstrated associations between rheumatoid arthritis and periodontal disease and these findings have been extensively reviewed [6]. Rheumatoid arthritis and periodontal disease share several pathobiological processes in common. These include similar cellular activity at inflammation sites, microenvironmental and serum cytokine profiles, matrix metalloproteinases and other mediators as well as osteoclast-mediated bone destruction [7]. Previous research has found periopathogenic bacteria in the synovium of rheumatoid arthritis patients, indicating that joint seeding and localized inflammatory amplification may be one of the reasons [8]. Other studies have identified common genetic risk factors, such as the Human Leukocyte Antigen (HLA)-DR shared epitope, polymorphisms and epigenetic modifications in cytokine genes for both the diseases. A recent study has also reported similar interleukin-6 promoter methylation in both rheumatoid arthritis and periodontal disease [9]. The severity of periodontal disease corresponds with the progression of rheumatoid arthritis. Clinical studies have additionally revealed that alveolar bone loss in rheumatoid arthritis patients with periodontal disease mirrors the erosions seen in other areas affected by rheumatoid arthritis [4]. In this present study we aimed to compare the effects of non-surgical periodontal therapy (NSPT) on gingival crevicular fluid levels and serum of omentin in periodontitis subjects with and without rheumatoid arthritis (RA). Poor periodontal health status was indicated by the RA + P groups where plaque index, modified gingival index, probing pocket depth and clinical attachment level increased. These results are consistent with earlier research by Ding *et al.* [5] and Gamel *et al.* [10]. In comparison to the chronic periodontitis-only (P), rheumatoid arthritis only (RA) and control groups, a study by Ding *et al.* revealed a significant difference in periodontal parameters and the serological indicators in the Rheumatoid arthritis and chronic periodontitis (CP) groups. Given that both rheumatoid arthritis and periodontitis include an inflammatory response brought on by the release of cytokines, this supports a connection between the two disorders. Signal transducer and activator of transcription 3 (STAT3) is stimulated by inflammatory processes, which might result in an immunological response that can affect periodontal health outcomes by causing periodontal damage. In addition to having periodontitis, rheumatoid arthritis patients frequently experience functional impairments in their upper limbs, which may have contributed to their general lack of manual dexterity and compromised dental hygiene [5]. Group P was found to have greater serum and gingival crevicular fluid omentin levels than the other groups. Dogan *et al.* [11] and Schäffler *et al.* [12] proved that the adipokine omentin has potential anti-inflammatory properties. Dogan *et al.* proposed that chronic periodontitis patients with and without diabetes mellitus had lower omentin levels in their gingival crevicular fluid level [11]. Zhang *et al.* also found that rheumatoid arthritis patients with Type 2 diabetes mellitus (T2D) had significantly reduced omentin expression in their synovial fluid [13].

Omentin levels in the synovial fluid of rheumatoid arthritis patients were found to be lower by Senolt *et al.* which may indicate that this adipokine may change as the disease progresses of rheumatoid arthritis [1]. Similarly, obesity and periodontitis were linked to lower omentin levels, according to Balli *et al.* Omentin may have an anti-inflammatory impact via blocking the cyclooxygenase-2 (COX-2) pathway that is triggered by TNF-α [14]. The AMP-activated protein kinase (AMPK) and endothelial nitric oxide synthase (eNOS)/NO pathways are further upregulated as a result of blocking JNK activation [15-16]. Omentin inhibits the extracellular regulated protein kinases (ERK)/NF-κB, which in turn down-regulates TNF-α-induced expression of intracellular adhesion molecule-1 (ICAM-1) and vascular cell adhesion molecule 1 (VCAM-1) [11]. Patients in Group P and RA+P underwent NSPT. NSPT is considered the goal standard treatment option for periodontal disease. To stop the disease from spreading, it is crucial to disrupt the oral biofilm that contains the pathogenic bacteria. In addition to preventing the growth of periodontal disease, this treatment also helps prevent the germs from entering the bloodstream, which lowers the load of inflammation throughout the body [17]. The patients were recalled after 6 weeks following NSPT and the periodontal parameters, serum as well as gingival crevicular fluid was re-collected for biochemical analysis. After NSPT (T1), the P & rheumatoid arthritis +P group's mean values for all periodontal parameters (plaque index, modified gingival index, probing pocket depth and clinical attachment level) decreased. The mean change in all the periodontal parameters from To to T1 was statistically significant. NSPT significantly improved clinical attachment level (CAL) and reduced probing pocket depth (PPD) in cases of moderate or severe periodontitis. Sanz *et al.* and Badersten *et al.* reports indicate that NSPT reduces the number of gingival sites that bleed during probing and facilitates a transition in oral microbiota from gram-negative to gram-positive bacteria [18-19]. Furthermore, Umeda *et al.* in their study indicated that NSPT decreases the number of pathogenic microorganisms including black-pigmented species and spirochetes while increasing coccoid cells [20]. These microbial changes are associated with improved clinical periodontal parameters, such as increased clinical attachment, reduced pocket depth and decreased inflammation. It has been demonstrated that adipokines, including omentin, which are secreted by adipose tissues, are important in the onset and advancement of periodontitis and rheumatoid arthritis [21]. After NSPT, the mean serum and gingival crevicular fluid levels of Omentin rose in the P and RA+P groups. Following NSPT, the patient's periodontal health status improved in tandem with higher salivary omentin-1 levels. This implies that omentin-1 may contribute to the immuno-pathogenesis of chronic periodontitis and may have an anti-inflammatory function in the disease [22].

Although there was an increase in serum and gingival crevicular fluid levels of Omentin, the mean change between T0 and T1 was not statistically significant. Periodontitis patients included in this study had stage III grade B periodontitis. These patients had probing pocket depth greater than or equal to 6mm, vertical bone loss and furcation involvement. Non-surgical periodontal therapy results in reduction of microbial load and improvement in clinical parameters however the total reduction in the pocket could not be attained as it is common that in deep pockets or in areas of furcation involvement residual pockets remain after non-surgical periodontal therapy [23]. Serum and gingival crevicular fluid levels of omentin in the P and RA+P groups were correlated with periodontal parameters using Karl Pearson. Serum and gingival crevicular fluid levels of omentin were positively correlated with all periodontal parameters. This showed that when serum and gingival crevicular fluid levels of omentin increased, the degree of periodontal inflammation decreased. Omentin appears to inhibit inflammation by blocking Jun N-terminal kinase (JNK) pathway [24]. Similarly, Ahuja *et al.* in their study concluded that NSPT was effective in improving clinical parameters, reducing serum leptin levels and also improving glycemic status in patients with chronic periodontitis and chronic periodontitis with T2DM [25]. Given the study's limitations, it is possible that RA and periodontitis may have an impact on serum omentin levels and gingival crevicular fluid. To further validate these findings, larger sample size research and surgical procedures are required in the future.

## Conclusion:

Omentin levels were found to be lowest in the periodontitis patients with rheumatoid arthritis which significantly improved following non-surgical periodontal therapy. Omentin can thus serve as a prognostic biomarker in patients with periodontitis and rheumatoid arthritis. This can further be targeted as an anti-inflammatory therapeutic agent to combat the effect of periodontitis and rheumatoid arthritis.

## Funding information:

The study received partial funding from Meenakshi Academy of Higher Education and Research University.

## Figures and Tables

**Table 1:** Mean and standard deviation of periodontal and biochemical parameters in Groups P, RA and RA+P at T0 (Baseline)

**Variables**	**Group**	**Mean±SD**	**p-Value**
Plaque Index	Group P	2.04±0.28	
	Group RA	1.75±0.30	0.001*
	Group RA+P	2.15±0.221	
Modified Gingival Index	Group P	2.14±0.30	
	Group RA	1.70±0.23	0.001*
	Group RA+P	2.28±0.15	
Probing Pocket Depth (mm)	Group P	5.59±0.54	
	Group RA	3.13±0.93	0.001*
	Group RA+P	6.09±0.48	
Clinical Attachment Level (mm)	Group P	6.06±1.03	0.001*
	Group RA	3.13±0.93	
	Group RA+P	6.45±0.68	
Serum Omentin (pg/mL)	Group P	24.58±2.12	0.001*
	Group RA	18.82±1.53	
	Group RA+P	13.22±2.12	
GCF omentin (pg/mL)	Group P	16.46±1.02	0.001*
	Group RA	12.17±0.68	
	Group RA+P	6.79±1.39	

**Table 2:** Mean and standard deviation of periodontal and biochemical parameters in Groups P and RA+P at T1 (after 6 weeks)

**Variables**	**Group**	**Mean±SD**	**p-Value**
Plaque Index	Group P	0.98±0.14	
	Group RA+P	1.08±0.63	0.03*
Modified Gingival Index	Group P	1.04±0.06	
	Group RA+P	1.08±0.60	0.208
Probing Pocket Depth (mm)	Group P	3.20±0.40	
	Group RA+P	3.34±2.30	0.001*
Clinical Attachment Level (mm)	Group P	3.50±0.80	0.786
	Group RA+P	3.58±0.45	
Serum Omentin (pg/mL)	Group P	27.84±2.87	0.09
	Group RA+P	30.20±3.33	
GCF omentin (pg/mL)	Group P	19.82±8.70	0.05*
	Group RA+P	25.25±5.32	

**Table 3:** Mean change in periodontal and biochemical parameters in Groups P and RA+P from To to T1

**Variables**	**Group**	**Mean±SD**	**p-Value**
Plaque Index	Group P	1.06±0.21	
	Group RA+P	1.07±0.20	0.001*
Modified Gingival Index	Group P	1.10±0.24	
	Group RA+P	1.20±0.15	0.001*
Probing Pocket Depth (mm)	Group P	2.38±0.39	
	Group RA+P	2.73±0.45	0.001*
Clinical Attachment Level (mm)	Group P	2.56±0.39	0.007*
	Group RA+P	2.87±0.72	
Serum Omentin (pg/mL)	Group P	-3.25±3.15	0.09
	Group RA+P	-16.90±4.23	
GCF omentin (pg/mL)	Group P	-3.36±9.64	0.274
	Group RA+P	-18.9±5.9	

**Table 4:** Correlation between mean change in periodontal and biochemical parameters in Group P

**Variables**		**Serum Omentin**	**GCF Omentin**
Plaque Index	Correlation coefficient	0.82	0.89
	p-Value	<0.01*	<0.01*
Modified Gingival Index	Correlation coefficient	0.81	0.91
	p-Value	0.03*	<0.01*
Probing Pocket Depth (mm)	Correlation coefficient	0.84	0.93
	p-Value	<0.01*	<0.01*
Clinical Attachment Level (mm)	Correlation coefficient	0.83	0.94
	p-Value	0.03*	0.01*

**Table 5:** Correlation between mean change in periodontal and biochemical parameters in Group RA+P

**Variables**		**Serum Omentin**	**GCF Omentin**
Plaque Index	Correlation coefficient	0.84	0.93
	p-Value	0.04*	<0.01*
Modified Gingival Index	Correlation coefficient	0.91	0.9
	p-Value	0.03*	<0.01*
Probing Pocket Depth (mm)	Correlation coefficient	0.84	0.86
	p-Value	0.03*	<0.01*
Clinical Attachment Level (mm)	Correlation coefficient	0.92	0.95
	p-Value	0.01*	<0.01*
